# Promoting responsive care and early learning practices in Northern Ghana: results from a counselling intervention within nutrition and health services

**DOI:** 10.1017/S1368980024000156

**Published:** 2024-02-08

**Authors:** Enam Aidam, Veronica Varela, Fauzia Abukari, Kelsey A Torres, Marie Paul Nisingizwe, Jennifer Yourkavitch, Eliasu Yakubu, Abdulai Abubakari, Rashida Ibrahim, Lesley Oot, Kathryn Beck, Selorme Azumah, Al-Hassan Issahaku, Joyce Apoassan Jambeidu, Lutuf Abdul-Rahman, Catherine Adu-Asare, Malia Uyehara, Kristen Cashin, Romilla Karnati, Catherine M Kirk

**Affiliations:** 1 USAID Advancing Nutrition, 2733 Crystal Drive, Fourth Floor, Arlington, VA 22201, USA; 2 JSI Research & Training Institute, 2733 Crystal Drive, Fourth Floor, Arlington, VA 22201, USA; 3 USAID Advancing Nutrition Ghana, Plot# 11, Jisonaayili-Gurugu, Tamale, Ghana; 4 University of British Columbia, School of Population and Public Health, 2206 East Mall, Vancouver, BC, Canada; 5 Results for Development, 1111 19th Street NW, Washington, DC, USA; 6 Saha Consulting and Services Limited, P. O. Box 430, Tamale, Ghana; 7 University for Development Studies, School of Public Health, Department of Global and International Health, P.O. Box TL1350, Tamale, Ghana; 8 Feed the Future Resilience in Northern Ghana Systems Strengthening, BA184 Dohana Kpema Street Gumani, Tamale, Ghana; 9 Abt Associates, 10 Fawcett Street, Cambridge, MA, USA; 10 United States Agency for International Development Ghana, No. 24 Fourth Circular Road, Cantonments, P.O. Box 1630, Accra, Ghana; 11 Ghana Health Service, DoDoo Lane, Osu, Accra, Ghana; 12 Save the Children US, 501 Kings Highway E, Fairfield, CT, USA; 13 ZemiTek LLC, USAID’s Global Solution Ventures, 1300 Pennsylvania Avenue NW, Washington, DC, USA

**Keywords:** Nurturing care, Early childhood development, Parenting, Responsive care, Early learning, Infant and young child feeding, Counselling

## Abstract

**Objective:**

This study assesses change in caregiver practices after integrating responsive care and early learning (RCEL) in nutrition and health services and community platforms in northern Ghana.

**Design:**

We trained health facility workers and community health volunteers to deliver RCEL counselling to caregivers of children under 2 years of age through existing health facilities and community groups. We assessed changes in caregivers’ RCEL practices before and after the intervention with a household questionnaire and caregiver–child observations.

**Setting:**

The study took place in Sagnarigu, Gushegu, Wa East and Mamprugu-Moagduri districts from April 2022 to March 2023. Study sites included seventy-nine child welfare clinics (CWC) at Ghana Health Service facilities and eighty village savings and loan association (VSLA) groups.

**Participants:**

We enrolled 211 adult caregivers in the study sites who had children 0–23 months at baseline and were enrolled in a CWC or a VSLA.

**Results:**

We observed improvements in RCEL and infant and young child feeding practices, opportunities for early learning (e.g. access to books and playthings) in the home environment and reductions in parental stress.

**Conclusions:**

This study demonstrates the effectiveness of integrating RCEL content into existing nutrition and health services. The findings can be used to develop, enhance and advocate for policies integrating RCEL into existing services and platforms in Ghana. Future research may explore the relationship between positive changes in caregiver behaviour and improvements in child development outcomes as well as strategies for enhancing paternal engagement in care practices, improving child supervision and ensuring an enabling environment.

The first 3 years of a child’s life are a crucial window of opportunity to support healthy brain development, protect children from adverse experiences and set the foundation for all future learning, behaviour and health^(1,2)^. An estimated 43 % of children under the age of 5 in low- and middle-income countries are at risk of not achieving their developmental potential and the proportion is even higher in Sub-Saharan Africa (66 %)^(3)^. The WHO, United Nations Children’s Fund (UNICEF), the World Bank and partners launched the Nurturing Care Framework in 2018 to promote the holistic care children need to improve early childhood development (ECD) outcomes, including good health, adequate nutrition, safety and security, responsive caregiving and opportunities for early learning^(4)^.

Ghana, a lower-middle-income country in Sub-Saharan Africa, has seen improvements in childhood outcomes over the last two decades; however, 23 % of children under 5 are at risk of not meeting their developmental potential due to stunting or extreme poverty, only 34 % of children receive early stimulation at home and caregivers want more support around ensuring optimal child development^(5,6)^. The Government of Ghana has made strong political commitments to improving children’s development since 2004 when they issued a multi-sectoral Early Childhood Care and Development (ECCD) Policy^(7)^, which has since been updated to align with the Nurturing Care Framework. In 2018, the Ministry of Gender, Children and Social Protection also developed ECCD Standards for children aged 0–3 years^(8,9)^.

The substantial body of evidence supporting nurturing care presents an opportunity to enhance childhood outcomes by integrating responsive care and early learning (RCEL) into existing nutrition and child health services^(10,11)^. USAID Advancing Nutrition – the Agency’s flagship multi-sectoral nutrition project that seeks to address the causes of malnutrition – developed the *RCEL Addendum* counselling package to complement UNICEF’s widely used 2013 *Community-Based Infant and Young Child Feeding (C-IYCF) Counselling Package* with these elements of nurturing care that are absent from the package^(12)^. The *RCEL Addendum* addresses some of the gaps in the C-IYCF package for nurturing care content, particularly around responsive care and feeding, early learning, child development and supporting children with feeding difficulties^(13)^.

Recent studies in Ghana have highlighted challenges with sustained uptake of RCEL practices and gaps in health services around ECD^(6,14–16)^. The purpose of this study is to evaluate the effectiveness of integrating RCEL content with infant and young child feeding (IYCF) counselling in nutrition and health service delivery and community-based platforms through assessing caregiver behaviours after programme implementation.

## Methods

### Study settings

**Fig. 1:**
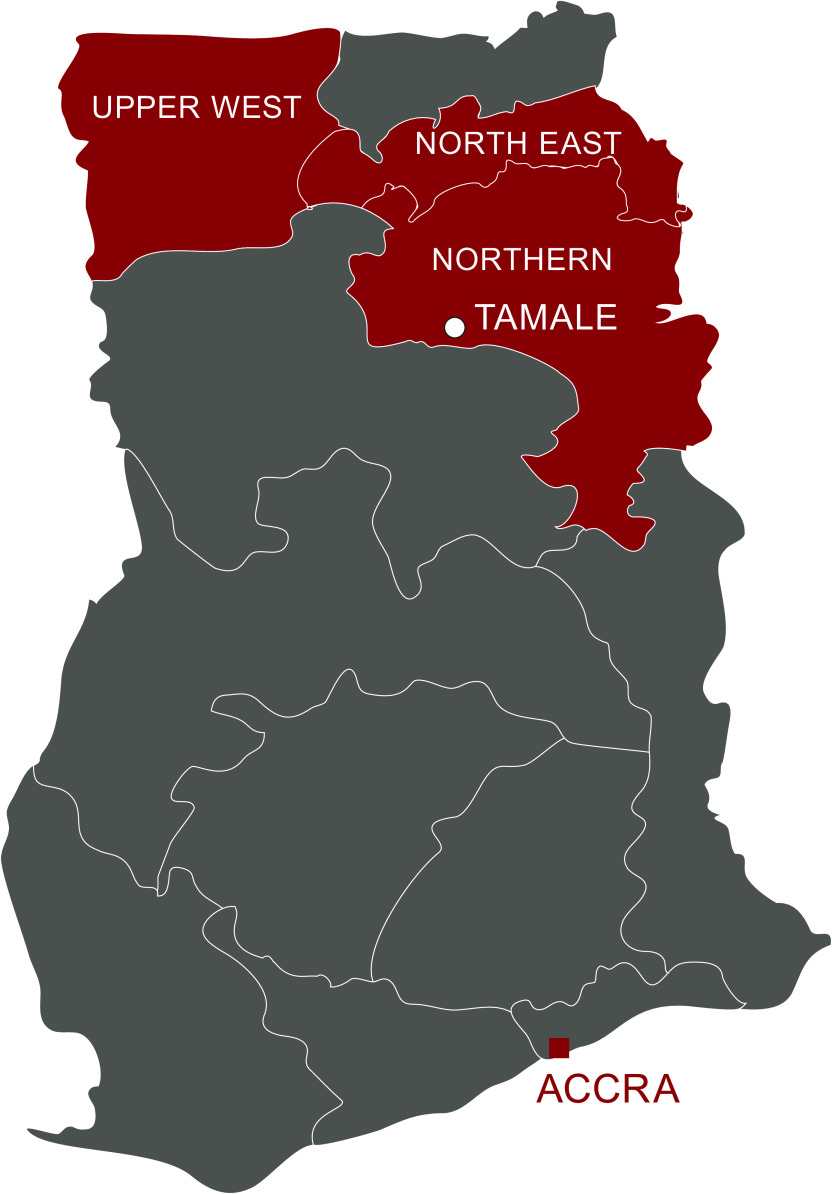
Map of study regions

The study took place across three regions of northern Ghana – Northern, Upper West and North East (Fig.1). Districts were selected based on the existence of ongoing IYCF intervention/activities by USAID Advancing Nutrition. One district was selected in each region in close consultation with regional and district-level health authorities. The Upper East region was originally included in the study; however, due to conflict in the selected district we replaced the selected district with a second district in the Northern region. The study included twenty-one communities, seventy-nine Ghana Health Service (GHS) facilities (health centres and community health planning and services compounds) and eighty village savings and loan associations (VSLA)^^1^^ across the four districts (Gushegu, Sagnarigu, Wa East and Mamprugu-Moagduri).

### Intervention

The *RCEL Addendum* package (implementation guidance, training materials and counselling cards) focuses on the following topics related to improving ECD outcomes: responsive care, responsive feeding, early learning, monitoring children’s development and caregiver well-being. The package also includes content related to supporting children with feeding difficulties, which was included as an observed gap in existing IYCF content, particularly for children with disabilities.

**Fig. 2:**
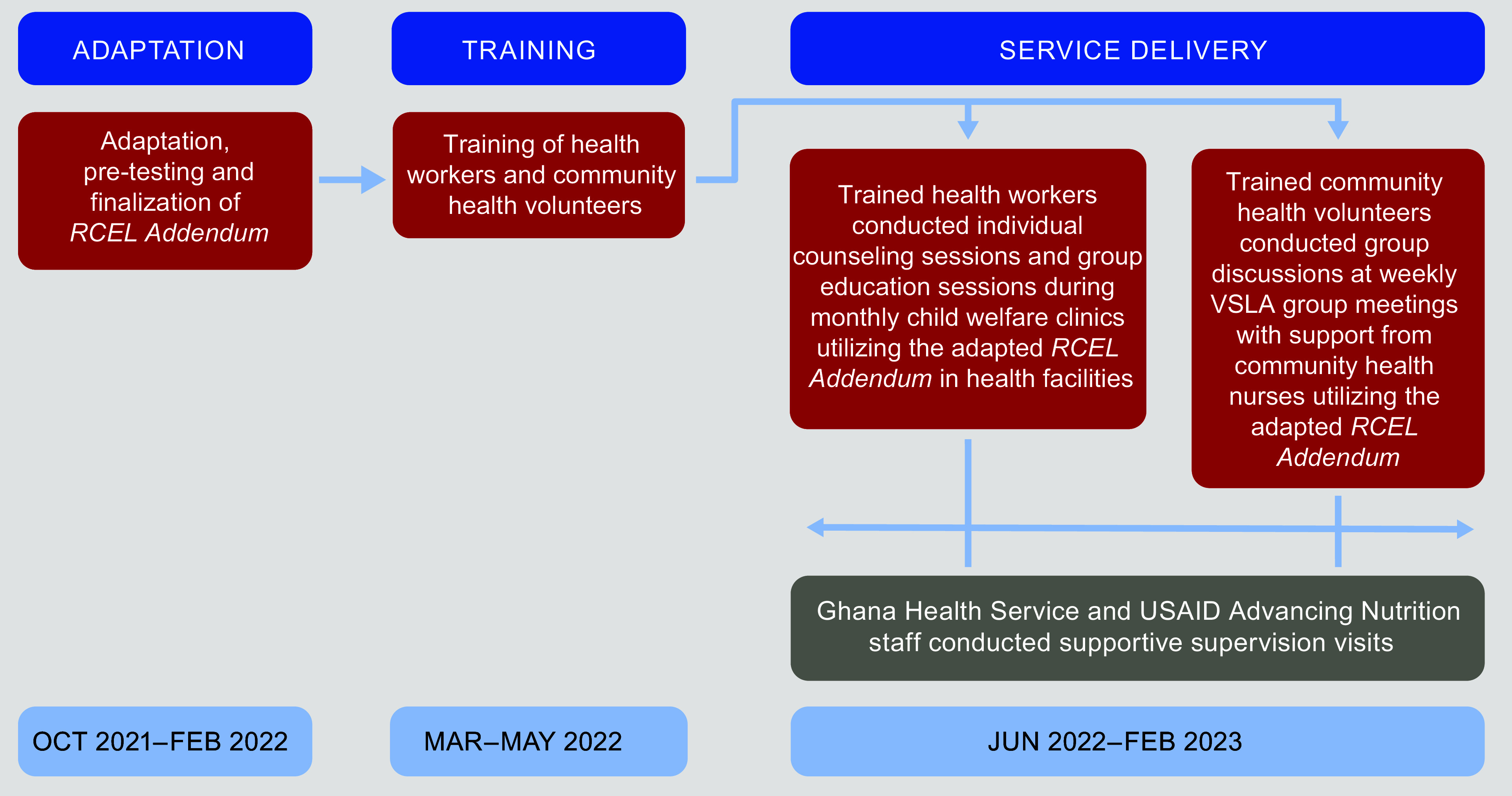
Intervention implementation approach

The intervention took place over 12 months, with 3 months of training from March to May 2022 followed by 9 months of service delivery from June 2022 to February 2023. It focused on using the *RCEL Addendum* counselling cards during contacts with caregivers of children 0–23 months including: (1) individual tailored counselling sessions (20–30 min) and group education sessions conducted by health workers at primary healthcare facilities during monthly child welfare clinics (CWC), following the GHS CWC existing protocols, and (2) group discussions facilitated by community health volunteers (CHV) with support from community health nurses at weekly VSLA meetings (Fig.2). CHV were selected for training from the twenty-one communities where USAID Advancing Nutrition was already supporting VSLA groups in each district. Working with GHS, CHV were purposely selected based on their activeness and involvement in existing community health activities. At least three CHV were selected from each community.

**Fig. 3:**
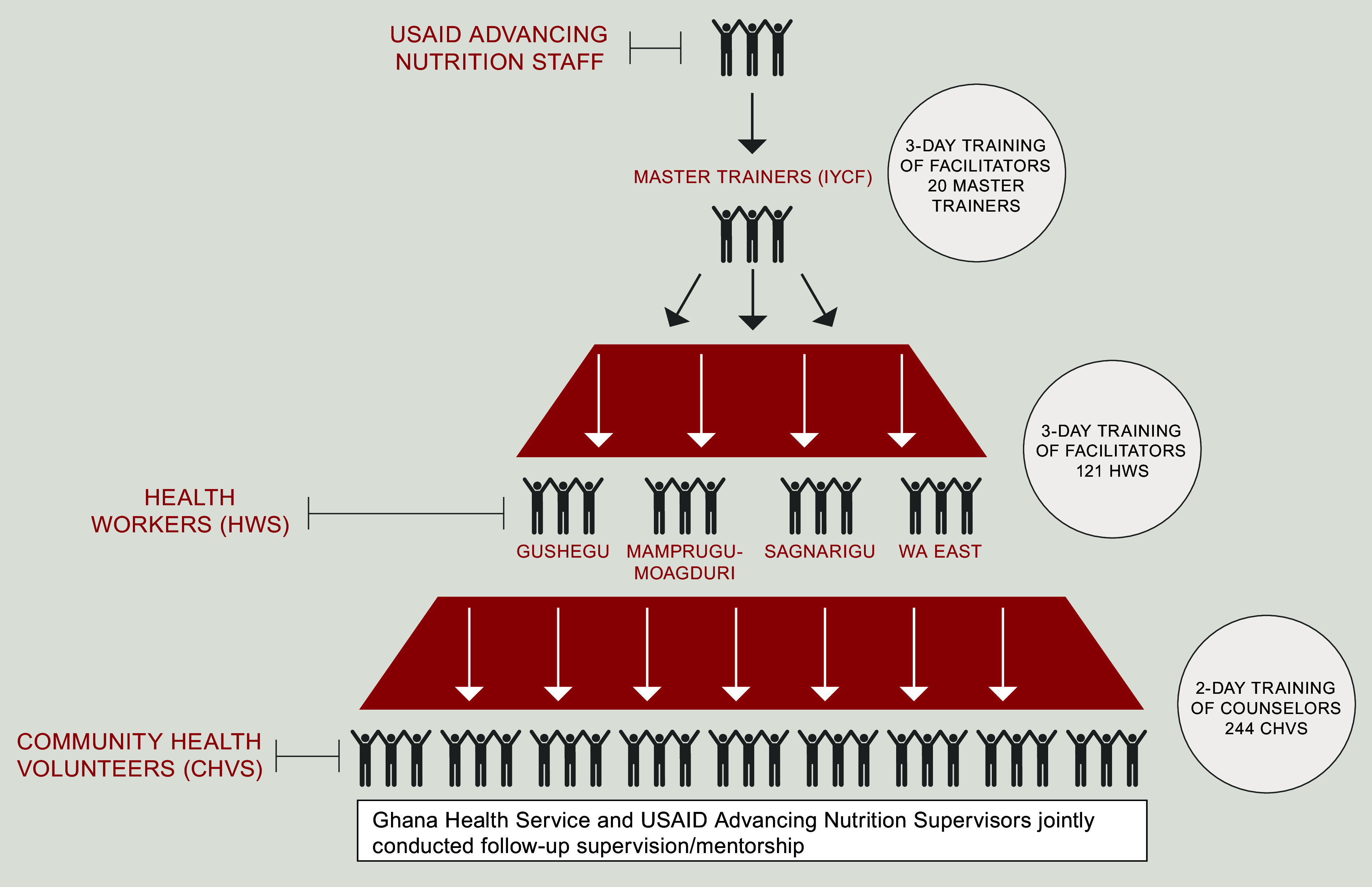
Cascade training approach

In accordance with the GHS cascade training approach (Fig.3), one national-level 3-d training was held for master trainers followed by four district-level (health worker) 3-d trainings for facilitators. Finally, eight 2-d training sessions for CHV were held. The training sessions reinforced essential counselling skills taught in IYCF trainings, introduced new RCEL content, and for the facilitator sessions, also oriented participants to supportive supervision and mentorship. As part of their training, all participants took a pre- and post-test to assess their knowledge gained. After the cascade trainings were completed, trained health workers and CHV used the counselling cards with caregivers. USAID Advancing Nutrition and GHS conducted at least two rounds of supportive supervision visits for trainees and provided on-the-job coaching and mentorship. Supervisors visited trainees and observed their interactions with caregivers utilising an adapted IYCF supportive supervision checklist. Supervisors observed the trainees and used the checklist to examine trainee competencies, including listening and learning skills, building confidence, providing support skills and using the 3-step counselling approach (i.e. assess, analyse and act) to deliver RCEL messages for both individual counselling and group sessions. As part of the visits, supervisors met with trainees after observations to discuss their results and opportunities for improvement.

### Study design

We assessed changes in caregivers’ RCEL, IYCF and child supervision practices, and stress levels through a survey and observations with approximately 12 months of intervention implementation using a pre–post design, with about 3 months of training followed by about 9 months of individual counselling and group sessions. Baseline and endline data were collected from 22 April to 2 May 2022 and 9 March to 17 March 2023, respectively. We completed all baseline data collection before the start of counselling and group sessions.

### Study population

We enrolled a single cohort of primary caregivers in the four study districts who were 18 years of age or older, had children 0–23 months at baseline and were enrolled in a CWC or members of a local VSLA. Participants provided informed consent to be included in the study.

### Sampling methods

We used convenience sampling to select the priority district, based on availability of other programmes in the district and a situational analysis conducted by USAID Advancing Nutrition^(15)^. We aimed to avoid districts with a high saturation of interventions and focused on areas where the situational analysis had been conducted to adapt the intervention based on the findings of the study.

**Fig. 4:**
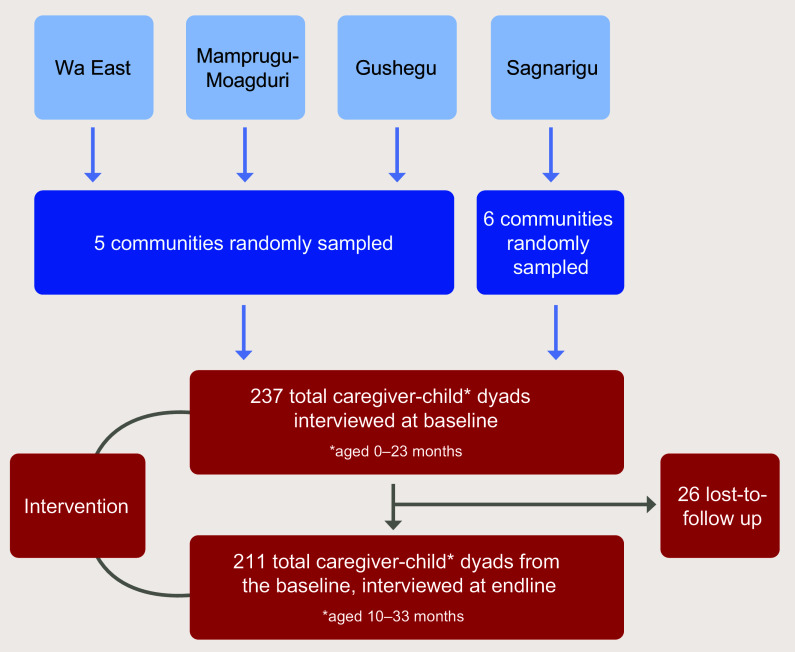
Study design

Five communities were randomly selected in three of the study districts (Gushegu, Wa East and Mamprugu-Moagduri), and six communities were randomly selected in the Sagnarigu District due to fewer caregivers per community, totalling twenty-one communities across the four study districts (Fig.4).

Initially, a list of caregivers of children aged 0–23 months was generated by the district health directorates from CWC registries at health facilities within each district. A list of eligible caregivers of children aged 0–23 months was also compiled from twenty VSLA group registries who were working with USAID Advancing Nutrition. These groups were supported and equipped with nutrition information and knowledge to improve household food and nutrition security in each study district. The two lists were cross referenced, and any duplicate entries were eliminated.

From the lists, we randomly selected twelve eligible caregivers from each community, with an additional three replacement caregivers if the original target mothers could not be reached or refused. In one community (Gbolo) where the sampling list had fewer than twelve mothers, additional mothers from a nearby community were randomly selected using the same procedure to reach the desired sample size.

Enumerators liaised with local CHV to identify and recruit other caregivers meeting the eligibility criteria using random sampling.

### Sample size

Sample size was calculated based on changes in responsive caregiving practices. Inferring expected change in measures of practices from a similar study in Bangladesh^(17)^, we estimated that a baseline sample size of 219 was required in order to detect a 10 percentage point change in RCEL practices from pre- to post-intervention with 80 % power, 95 % confidence and an assumption of 10 % loss to follow-up as well as a design effect of 2 and a finite population adjustment. In total, we recruited and interviewed 237 caregiver–child dyads for the baseline survey. We recorded a loss-to-follow up of twenty-six respondents (11 %), resulting in 211 respondents at the endline (Fig.4).

### Data collection

The data collection team comprised twelve trained enumerators and four supervisors fluent in English and local languages. The data collection team for the baseline were largely the same at the endline. Training on the study tools and processes was conducted in English at baseline and endline, including close review of the study tools for translation of key words by enumerators into the appropriate dialect of the respondent following standard approaches^(18)^. Data collectors translated from English to the respondents’ language at the point of data collection and used Kobo Collect to record data at the participants’ homes. Survey questions and response options were programmed into the digital format in English incorporating data entry restrictions and skip patterns to ensure data completeness and accuracy. The survey was piloted in two of the study districts (Sagnarigu and Wa East) outside of the selected study communities in March 2022.

#### Primary outcomes

Responsive caregiving was assessed using a new tool developed by the Harvard T.H. Chan School of Public Health, which was validated in low-resource settings in Pakistan in 2021 (not yet published)^(19)^. The tool applies a structured coding process to observations of 5-minute play interactions between a caregiver and child with a novel stimulus (locally available toy or picture card) provided by the study team. Trained enumerators observe a 5-minute play interaction and tally the number of responsive interactions (i.e. child-initiated), caregiver-initiated interactions or negative interactions and note if they were verbal or non-verbal. The percentage of each type of interaction (responsive, caregiver-initiated or negative) was calculated using the denominator of all of the observed interactions during the 5-minute period^(19)^. The tool was pre-tested for use in Ghana and then enumerators were trained on the tool and validated upon ensuring a minimum of 0·6 inter-rater reliability; observations were conducted by a limited number of enumerators to ensure quality and standardisation.

Early learning was assessed using a new 14-item early learning measure of play materials and interactions with caregivers in the prior 24 h^(19)^ and the Multiple Indicator Cluster Survey (MICS) Family Care Indicators^(20)^. Family Care Indicators were calculated following standard definition on the number of stimulating engagement activities (reading books or stories, songs, play activities and objects) that an index child was engaged in with adult household members in the last 3 d.

#### Secondary outcomes

To assess IYCF outcomes, the study team calculated three indicators for infants and young children aged 6–23 months as defined by UNICEF and WHO’s Technical Expert Advisory Group on Nutrition Monitoring^(21)^ using a 24-h dietary recall: minimum meal frequency, minimum dietary diversity and minimum acceptable diet. Responsive feeding indicators are currently not a standard component of Demographic and Health Surveys, so they were not included in the assessment.

Caregivers’ stress levels associated with parenting were assessed using the Parenting Stress Index Short Form on five indicators, each distinctively calculated as a sum score of different components of a 35-item Likert scale questionnaire^(22)^. The indicators included ‘parental distress’ (PD), ‘parent–child dysfunction’ (P-CDI), ‘difficult child’ (DC), ‘mean total stress’ scores and the percentage of caregivers reporting high parental stress calculated as sum of scores in the 85th percentile or higher.

Additionally, we calculated whether a child was inadequately supervised (defined as a child left alone at home or under the supervision of a child under 10 years for at least an hour or more in the prior week). We measured supervision using the UNICEF MICS (2020) question on adequate supervision^(20,23)^.

#### Programme exposure

Programme exposure was measured at baseline and endline. Caregivers were asked how many times they visited a health facility or participated in VSLA group meetings to discuss their child’s development in the past 6 months. Programme exposure was then calculated by summing the number of times a caregiver visited a health facility and the number of times a caregiver participated in the VSLA group meetings to discuss their child’s development in the past 6 months. We collected routine monitoring data quarterly for donor reporting. The data were inclusive of the number of children under 5 years old reached with nutrition interventions at health facilities with health workers trained on RCEL and the number of individuals participating in VSLA group meetings with trained community health workers. These data showed no large increases or decreases in children reached or individuals participating in the VSLA groups throughout programme implementation. However, due to missing data we did not have complete programme exposure data at the endline. Therefore, given the consistent counts of children reached and individuals participating in VSLA groups throughout programme implementation in the routine monitoring data, we assumed baseline programme exposure stayed consistent throughout programme implementation and used baseline programme exposure values for our analysis (see online supplementary material, Supplementary Material 1).

#### Additional socio-demographic indicators

In addition to standard demographic measures such as education and age, screen exposure was measured utilising an adapted questionnaire from the Seven-in-Seven Screen Exposure Questionnaire^(24)^. The questionnaire included five items: daily screen time, viewing with parent(s), setting screen limits, screen exposure during meals and age of onset of screen exposure. Each item was scored from 0 to 3, with 0 being low exposure and 3 being high exposure. Total screen exposure was calculated on a scale of 0–13 based on the sum of scores of the five items.

Caregiver’s functioning was measured using the Washington Group Short Set on Functioning threshold for identifying potential disability (‘a lot of difficulty’ or ‘cannot do at all’)^(25)^.

### Data analysis

We used StataMP (version 17, StataCorp) for analyses, including caregiver–child pairs that completed both baseline and endline surveys and observations. We calculated descriptive summary statistics for the index child (age, sex) and caregiver (age, sex, level of education, literacy level, marital status), and primary and secondary outcome measures. The change in these indicators from baseline to endline was analysed using paired t-tests for continuous variables or McNemar’s test for paired proportions for categorical variables. We conducted bivariate regression analyses to examine associations of prioritised factors, based on existing literature, with several outcomes: caregiver–child interactions that were responsive; children with whom adult household members have engaged in four or more activities; the number of stimulating engagement activities by a caregiver with objects (e.g. playthings) and/or people (adults and peers); caregiver–child interactions that are negative; caregivers reporting high parental stress; children left with inadequate supervision in the past week and children 6–23 months who are achieving a minimum acceptable diet. The bivariate analyses included the following prioritised baseline factors: caregiver education, whether the child’s father was living in the home, child sex, screen exposure, child’s age, mother’s age and the number of household members. We then controlled for all factors that had a *P*-value less than 0·20 in multivariable regression models to assess the association of programme exposure with the outcomes. Additionally, we controlled for child age, caregiver education and the baseline measure of the outcome of interest in all models. We used inverse probability of treatment weights to control for the identified factors in statistical models for all outcomes except for the number of negative interactions. For this specific outcome, we directly included those factors in the regression equation due to the inability to calculate inverse probability of treatment weight. We trimmed the inverse probability of treatment weight to exclude extreme outliers, removing 5 % of the total sample for each model, which improved model performance.

To further assess if changes from baseline to endline were a result of children getting older, we conducted a sensitivity analysis to examine changes in all primary and secondary outcomes between children who were 12–23 months at baseline (*n* 66) and children who were 12–23 months at endline (*n* 151). We used unpaired t-tests and two sample tests of proportions to assess the differences between indicators at the two time points.

## Results

### Demographics and programme exposure

Children’s mean age was 9·2 months (sd 5·7) at baseline and 19·7 months (sd 5·8) at endline (Table 1). Almost all caregivers (98·6 %) were the biological mother of the index child and were married (95·7 %). The mean age of mothers and fathers at baseline was 29·5 and 39·8 years, respectively.

**Table 1:** Participant demographics at baseline and programme exposure

Demographics	***n***	%
Region		
Northern	114	54·0
North East	50	23·7
Upper West	47	22·3
Child’s sex		
Male	109	51·7
Female	102	48·3
Child’s age		
0–5 months	65	30·8
6–11 months	80	37·9
12–17 months	43	20·4
18–23 months	23	10·9
Caregiver’s relationship to child		
Biological mother	208	98·6
Aunt/uncle	1	0·5
Grandparent	2	1·0
Caregiver marital status		
Single	4	1·9
In a partnership/living together	5	2·4
Married	202	95·7
Caregiver education		
Primary completed	47	22·3
Secondary or higher completed	53	25·1
None completed	111	52·6
Caregiver literacy		
Not able to read and write	165	78·2
Able to read and write	46	21·8
Caregiver functioning		
Has a disability	12	5·7
No disability	199	94·3
Child’s father living in the home		
No	22	10·7
Yes	184	89·3
Screen time use		
Child uses screen device	133	63·0
High level of screen exposure	21	15·8
	Mean	s d
Household size		
Number of people in the household	12·4	8·6
Number of children under 18 years	4·6	3·0
Number of children under 2 years	1·2	0·6
Child age at baseline	9·2	5·7
Child age at endline	19·7	5·8
Mother’s age	29·5	6·2
Father’s age	39·8	14·6
Participated in the VSLA group meetings to discuss child’s development in the previous 6 months	1·9	4·6
Visited a health facility to talk with a health worker about child’s development in the previous 6 months	1·9	2·1
Participated in the VSLA group meetings and/or visited a health facility to discuss child’s development in the previous 6 months	3·9	5·7

The majority of caregivers (52·6 %) had not completed primary school, while 22·3 % completed at least a primary level and 25·1 % completed secondary school or higher. Given the study was largely in rural areas, most caregivers (78·2 %) could not read and write. Few caregivers (5·7 %) had a disability. At baseline, households had an average of twelve members with an average of five children under 18. Most fathers lived in the home with the index child (89 %). Regarding programme exposure, respondents reported participating in a VSLA group meeting or visiting a health facility to discuss their child’s development an average of two times each in the past 6 months. Overall, participants reported participating in the VSLA group meetings and/or visiting a health facility to discuss their child’s development in the previous 6 months an average of four times.

### Differences from baseline to endline

#### Primary outcomes

All responsive care indicators had significant positive changes from baseline to endline in the paired comparison. There was a 46·3 percentage point increase in the mean number of caregiver–child interactions that were responsive to the child’s cues (*P* < 0·001) and a resulting 44·2 percentage point decrease in caregiver-initiated interactions (*P* < 0·001). Additionally, verbal caregiver–child interactions increased 5·6 percentage points (*P* < 0·001), and negative interactions decreased by 2·1 percentage points (*P* < 0·001). Similarly, all these positive changes, except for the increase in verbal interactions, remained statistically significant in the sensitivity analysis comparing children aged 12–23 months at baseline and endline (see online supplementary material, Supplementary Material 2).

All early learning indicators also had statistically significant, positive changes from baseline to endline. Notably, there was a 50·2 percentage point increase in the number of children with whom adult household members have engaged in four or more activities (*P* < 0·001). We also saw a statistically significant increase in the age sensitivity analysis. Increases in activities with both mothers (37·0 percentage points) and fathers (7·6 percentage points) were observed (*P* < 0·001 for both). All indicators of opportunities for engagement also showed significant improvements. There was a 52·6 percentage point increase in the number of children who played with homemade toys (*P* < 0·001) and a 49·3 percentage point increase in the number of children who play with two or more types of playthings (*P* < 0·001). Finally, the number of stimulating engagement activities by a caregiver with a child in the last 24 h significantly increased by 4·6 activities (*P* < 0·001). We also saw a statistically significant increase in all of these outcomes in the age sensitivity analysis.

#### Secondary outcomes

We found statistically significant increases in minimum dietary diversity (43·0 percentage points, *P* < 0·001) and minimum acceptable diet in children 6–23 months (27·9 percentage points, *P* < 0·001), from baseline to endline (Table 2), but only the increase in minimum dietary diversity was statistically significant in the age sensitivity analysis.

**Table 2:** Paired differences from baseline to endline

Paired differences							
Indicator	Total *n*	Baseline		Endline		Change from baseline to endline	
		% or mean (sd)		% or mean (sd)		Percentage point change or unit change	*P*
Caregiver–child interactions that are responsive to the child’s cues	211	15·1	17·2	61·4	18·4	46·3	<0·001
Caregiver–child interactions that are initiated by the caregiver	211	81·6	10·6	37·4	18·4	−44·2	<0·001
Caregiver–child interactions that are negative	211	3·3	10·1	1·2	4·7	−2·1	0·004
Caregiver–child interactions that are verbal	211	40·2	16·3	45·8	12·2	5·6	<0·001
Children with whom adult household members have engaged in four or more activities	211	15·2		65·4		50·2	<0·001
Number of activities with adult household members	211	2·8	1·1	4·0	1·4	1·2	<0·001
Children with whom fathers have engaged in four or more activities	211	1·0		8·5		7·6	<0·001
Number of activities with fathers	211	0·6	1·0	1·0	1·5	0·4	0·002
Children with whom mothers have engaged in four or more activities	211	5·2		42·2		37·0	<0·001
Number of activities with mothers	211	2·3	1·1	3·1	1·6	0·8	<0·001
Children who have three or more children’s books	211	1·0		4·7		3·8	0·039
Children who play with homemade toys	211	24·6		77·3		52·6	<0·001
Children who play with household objects/objects found outside	211	45·5		87·2		41·7	<0·001
Children who play with toys from a shop/manufactured toys	211	38·9		55·5		16·6	<0·001
Children who play with two or more types of playthings	211	30·3		79·6		49·3	<0·001
Number of stimulating engagement activities by a caregiver with a child from 0 to 23 months with objects (e.g. playthings) and/or people (adults and peers)	211	3·4	2·6	8·0	2·9	4·6	<0·001
Parental distress sub-scale (PD) score	211	34·4	9·0	30·9	10·4	−3·5	<0·001
Parent–child dysfunctional interaction (P-CDI) sub-scale score	211	32·4	5·2	30·3	5·8	−2·1	<0·001
Difficult child (DC) sub-scale score	211	31·3	6·4	29·5	7·6	−1·8	0·003
Total stress score	211	98·1	16·5	90·7	19·2	−7·4	<0·001
Caregivers reporting high parental stress	211	26·5		12·3		−14·2	<0·001
Left alone in the past week	211	34·1		41·7		7·6	0·109
Left under the supervision of another child younger than 10 years of age in the past week	211	18·0		44·1		26·1	<0·001
Left with inadequate supervision in the past week	211	38·4		64·5		26·1	<0·001
Children 6–23 months who are achieving minimum dietary diversity	93	30·1		73·1		43·0	<0·001
Children 6–23 months who are achieving minimum meal frequency	93	67·7		65·6		−2·1	0·732
Children 6–23 months who are achieving minimum acceptable diet	93	26·9		54·8		27·9	<0·001

All parental stress indicators improved from baseline to endline, and the percentage of caregivers reporting high stress decreased by 14·2 percentage points (*P* < 0·001). All of the improvements (decreases) in parental stress were also statistically significant in the age sensitivity analysis. We found a statistically significant increase in the percentage of children who were left with inadequate supervision in the past week (26·1 percentage points, *P* < 0·001); this change in supervision was also statistically significant in the sensitivity analysis (see online supplementary material, Supplementary Material 2).

### Associations between programme participation and outcomes

Factors that were significant at *P* < 0·20 included: child sex, child’s screen exposure, mother’s age and number of household members (see online supplementary material, Supplementary Material 3) and these were controlled for in models to examine the associations between programme participation and the outcomes.

Table 3 presents the adjusted analyses assessing the relationships between exposure to the programme and the outcomes of interest. Increased programme exposure (i.e. more sessions attended) was associated with a 28 % decrease in parental stress (relative risk (RR): 0·72, 95 % CI 0·66, 0·78), controlling for child age, child sex, caregiver’s education, mother’s age, number of household members and screen exposure. After controlling for child age, caregiver’s education and number of household members, there was a statistically significant unit increase of 0·11 in the number of stimulating engagement activities by a caregiver, with each additional programme session attended (95 % CI 0·02, 0·21). Additionally, there was a likely association with increased programme exposure and a slight increase in the likelihood of adult household members engaging in four or more activities with a child (RR: 1·04, 95 % CI 1·00, 1·07, *P* = 0·05). Programme exposure was not associated with any other outcomes of interest.

**Table 3:** Multivariable regression analysis of caregiver’s programme exposure and outcomes of interest

	Children with whom adult household members have engaged in four or more activities‡ (*n* 201)		Caregivers reporting high parental stress§ (*n* 133)		Children left with inadequate supervision in the past week|| (*n* 201)		Children 6–23 months who are achieving minimum acceptable diet‡ (*n* 88)		Number of caregiver–child interactions that were responsive|| (*n* 201)		Number of stimulating engagement activities by a caregiver¶ (*n* 201)		Number of caregiver–child interactions that are negative††(*n* 208)	
Programme exposure†	RR	95 % CI	RR	95 % CI	RR	95 % CI	RR	95 % CI	RD	95 % CI	RD	95 % CI	RD	95 % CI
	1·04	1·00–1·07	0·72*	0·66–0·78	1·02	0·98–1·05	1·02	0·97–1·09	0·75	–0·91–2·42	0·11*	0·02–0·21	−0·04	–0·16–0·07

RR, relative risk; RD, risk difference.

* Significant at *P* < 0·05.

† Programme exposure was defined as the number of times a caregiver visited a health facility or participated in VSLA group meetings to discuss their child’s development.

‡ Controlled for baseline measure, child age, child sex and caregiver’s education.

§ Controlled for baseline measure, child age, child sex, caregiver’s education, mother’s age, number of household members and screen exposure.

|| Controlled for baseline measure, child age and caregiver’s education.

¶ Controlled for baseline measure, child age, caregiver’s education and number of household members.

†† Controlled for baseline measure, child age, child sex, caregiver’s education and mother’s age.

## Discussion

Our intervention aimed to integrate RCEL content with counselling on IYCF topics through individual counselling and group discussions at primary healthcare facilities and community groups to improve caregiver RCEL practices. This study demonstrates the effectiveness of the integrated approach as we found increases in responsive care, early learning and complementary feeding practices. These increases held when we examined outcomes for the same ages to control for natural ageing effects associated with child learning and independence. We also observed an association between the number of counselling sessions attended and the number of engaging activities, controlling for demographic factors, indicating that exposure to the intervention positively influenced caregivers’ engagement practices.

One of the key strengths of this intervention lies in its integration with existing nutrition and child health services. There are numerous documented benefits of integrating nutrition and caregiving interventions, especially in the critical first 1000 d of a child’s life. Results of this study align with results from a study in India where the routine Integrated Child Development Services were enhanced with either complementary feeding support or complementary feeding, responsive care and play^(26)^. A similar intervention in Bangladesh that integrated a daily micronutrient supplementation with regular counselling sessions with peer educators on responsive feeding and play also reported a significantly increased change in mothers’ responsive behaviours and identification of various opportunities for stimulation in the home^(17)^. It is therefore not surprising that our intervention also demonstrated a similar pattern with an increase in not just the RCEL indicators but also an increase in two out of the three IYCF indicators. Increases in these practices are linked to improvements in ECD outcomes^(27)^; however, further study is required to know whether the *RCEL Addendum* intervention may go beyond changes in practices and contribute to improved ECD outcomes.

Our pre–post comparisons indicated statistically significant improvements in all RCEL practices and the home environment (e.g. improved access to books and toys around the home), as well as decreases in parenting stress. These promising findings align with other studies that have shown that regular contacts (e.g. at least once every six weeks) through the health system were sufficient to see positive changes in caregiver practices such as positive discipline, caregiver–child interactions and quality of stimulation in the home, or ECD outcomes^(28–31)^. This relatively light-touch approach may be more feasible in overcoming health system constraints such as limited staff time that have hindered implementation elsewhere^(32)^. In order to ensure sustained uptake of these practices, they should be monitored to know what additional support for which specific audiences (e.g. fathers, grandparents, young mothers) is needed. In particular, despite improvements in paternal engagement over the course of the intervention, at the endline only 8·5 % of children had fathers engage in four or more activities over the past 3 d; more work is needed to improve paternal engagement in care practices. Caregivers may also benefit from complementary activities (e.g. community dialogues, media campaigns, policy advocacy) that serve to ensure an enabling environment for the optimal practices.

Our close collaboration with GHS and alignment with existing materials (e.g. Maternal and Child Health Record Book), personnel, CWC and VSLA groups were integral to the intervention and may have had an amplification effect, reinforcing content from various health system contacts, specifically around developmental milestones. Our intervention also aligned with GHS’s common cascade training and supportive supervision approach, so trainees likely had familiarity with techniques, which facilitated uptake of the content. The training bolstered best practices for quality counselling and group facilitation, and this was a key area of focus during supervision.

We rolled out the programme in a stepwise manner while monitoring and supervising implementation in collaboration with GHS supervisors. This arrangement, which utilised existing supervisors, provided several opportunities for on-the-job coaching and mentoring, as well as addressing challenges that arose. A supportive supervision strategy was also employed by Aftab *et al.* during a randomised controlled implementation trial in Pakistan^(33)^. The study found that consistent, frequent and quality supervision coupled with refresher training led to improved performance outcomes of community health workers. Additionally, an evaluation conducted of the Nigeria C-IYCF counselling package specifically found supportive supervision was useful to strengthen counsellors’ provision of quality IYCF counselling and in turn improve IYCF outcomes^(34)^. Our intervention also leveraged existing touchpoints with caregivers through the health system and community groups, supporting both health workers and CHV to lead discussion around the RCEL content. Similar ECD interventions have also relied on trained community health workers or volunteers, with positive outcomes, given their trusted role in the community^(28,31,35,36)^.

The significant increase in children with inadequate supervision that we observed may be attributed to the natural ageing of children over the course of the study (average age 9 months at baseline, 19 months at endline), leading caregivers to feel more comfortable leaving them in the care of their older siblings. Iwo *et al*. found a similar pattern of children in the 0–4 years age group being left home alone in their analysis of Ghana MICS data^(37)^. The *RCEL Addendum* was not explicitly designed to address safety and security in a deliberate effort to focus efforts on the RCEL components of nurturing care and avoid overwhelming caregivers. However, the issue of inadequate supervision is common among Ghanaian communities and should be addressed with more focused efforts in the future. In northern Ghana in particular, as children age, caregivers may be more likely to leave them without adequate supervision as they attend to other activities^(37)^. This may be due to communal and kin-based living arrangements that are common among ethnic groups in the region with caregivers potentially leaving their children without direct adult supervision^(37,38)^. Furthermore, high rates of poverty in the north may result in prioritisation of work outside the home^(39–41)^. Rural northern communities are predominantly reliant on subsistence agriculture^(42)^. Given the baseline and endline surveys were completed during the farming season, as children got older, they may have been left in the care of their older siblings as the caregiver attended to farming activities.

This study had a few limitations. First, because the pre- and post-study design did not include a comparison group, we cannot determine whether the observed changes are directly attributable to the *RCEL Addendum* intervention. All study districts were in northern Ghana, a largely rural area heavily reliant on subsistence agriculture, which may limit generalisability of the findings to other contexts. It is important to acknowledge the Hawthorne effect may have influenced caregivers to practice more socially desirable behaviours. To minimise this potential bias, researchers were trained in appropriate observation techniques and ensured observation was done in a private area. We did not assess caregiver depression, which has been shown to have substantial impacts on care practices and child health and development outcomes^(43–49)^. Additionally, we did not measure changes in responsive feeding practices, which is a topic included in the RCEL counselling package and can impact IYCF practices due to the lack of metrics in common, large, population-based surveys, such as Demographic and Health Surveys, which are commonly used to measure health outcomes in low- and middle-income countries. Finally, we were not able to effectively measure programme exposure at the endline so we used baseline data to assume a constant rate of exposure based on programme monitoring data showing a consistent trend. Based on our findings and limitations, future research could include the addition of specific indicators to measure impacts of *RCEL Addendum* counselling on responsive feeding practices, which is an important integrated IYCF and responsive care practice. Additionally, areas of future research could include assessing the impact of the *RCEL Addendum* on ECD outcomes specifically and evaluating the implementation of the *RCEL Addendum* in emergency contexts.

### Conclusion

Working through existing health system structures and in close collaboration with the Government of Ghana, the integration of the *RCEL addendum* into IYCF counselling supported health workers and CHV to provide counselling and education to caregivers on RCEL practices. These findings can be used to develop, enhance or advocate for activities, programmes and policies to promote ECD integration into existing services and platforms in Ghana, and more broadly, that may be scalable and create an enabling environment for sustained uptake of practices. While it is expected that positive changes in behaviour translate to an improvement in children’s development, this is an area for future research. Subsequent studies in Ghana may consider exploring strategies for paternal engagement in care practices and improving child supervision.

## Supporting information

Aidam et al. supplementary material Aidam et al. supplementary material

## Footnotes

1 VSLA are predominately mothers’ groups widely used in Ghana to strengthen financial literacy and expand economic opportunity. USAID Advancing Nutrition leveraged these groups to share and discuss nutrition topics.

## Supplementary material

For supplementary material accompanying this paper visit https://doi.org/10.1017/S1368980024000156

## References

[1] Black MM , Walker SP , Fernald LCH et al. (2017) Early childhood development coming of age: science through the life course. Lancet 389, 77–90. 27717614 10.1016/S0140-6736(16)31389-7 PMC5884058

[2] Lo S , Das P & Horton R (2017) A good start in life will ensure a sustainable future for all. Lancet 389, 8–9. 27717611 10.1016/S0140-6736(16)31774-3

[3] Lu C , Black MM & Richter LM (2016) Risk of poor development in young children in low-income and middle-income countries: an estimation and analysis at the global, regional, and country level. Lancet Glob Health 4, e916–e922. 27717632 10.1016/S2214-109X(16)30266-2 PMC5881401

[4] World Health Organization, United Nations Children’s Fund & World Bank Group (2018) Nurturing Care for Early Childhood Development: A Framework for Helping Children Survive and Thrive to Transform Health and Human Potential. Geneva: World Health Organization.

[5] UNICEF (2021) Country profiles for early childhood development. Available at https://nurturing-care.org/wp-content/uploads/2023/10/ECD_Countdown2030_Global.pdf (accessed November 2023).

[6] Singh A , Torres KA , Maharjan N et al. (2023) Learning from health system actor and caregiver experiences in Ghana and Nepal to strengthen growth monitoring and promotion. PLoS ONE 18, e0282807. 36893119 10.1371/journal.pone.0282807 PMC9997959

[7] Republic of Ghana Ministry of Women and Children’s Affairs (2004) Early Childhood Care and Development Policy. Accra: Ministry of Women’s and Children’s Affairs.

[8] Caldwell K , Gow J , Karnati R & Manji S (2021) Promoting nurturing care within the health sector. Available at https://nurturing-care.org/wp-content/uploads/2021/03/Country_Brief_Ghana.pdf (accessed December 2023).

[9] Ministry of Gender, Children, and Social Protection (2018) Early childhood care and development standards (0–3 years). Available at https://www.unicef.org/ghana/media/2031/file/Early%20Childhood%20Care%20and%20Development%20Standards.pdf (accessed August 2023).

[10] Britto PR , Lye SJ , Proulx K et al. (2017) Nurturing care: promoting early childhood development. Lancet 389, 91–102. 27717615 10.1016/S0140-6736(16)31390-3

[11] Dulal S , Prost A , Karki S et al. (2021) Characteristics and effects of integrated nutrition and stimulation interventions to improve the nutritional status and development of children under 5 years of age: a systematic review and meta-analysis. BMJ Glob Health 6, e003872. 10.1136/bmjgh-2020-003872 PMC8319976 34321232

[12] Hromi-Fiedler AJ , Pérez-Escamilla R , Segura-Pérez S et al. (2022) Assessing the nurturing care content of UNICEF’s community infant and young child feeding counselling package: gaps, best practices, and lessons learned. Curr Dev Nutr 6, nzac018. 35368736 10.1093/cdn/nzac018 PMC8967086

[13] World Health Organization, United Nations Children’s Fund & World Bank Group (2023) Nurturing Young Children through Responsive Feeding. Geneva: World Health Organization.

[14] Sandow A , Tice M , Pérez-Escamilla R et al. (2020) Facilitators of responsive feeding/parenting knowledge and practices among parents in the central region of Ghana. Curr Dev Nutr 4, 1069.

[15] USAID Advancing Nutrition (2022) Situational Analysis of Early Childhood Care and Development Services in Ghana: Final Report. Arlington, VA: USAID Advancing Nutrition.

[16] USAID Maternal and Child Survival Program (MCSP) (2021) Ghana: Promoting nurturing care within the health sector. Available at https://nurturing-care.org/ghana-promoting-nurturing-care/ (accessed May 2023).

[17] Aboud FE & Akhter S (2011) A cluster-randomized evaluation of a responsive stimulation and feeding intervention in Bangladesh. Pediatr 127, e1191–e1197. 10.1542/peds.2010-2160 21502222

[18] Logan C (2018) 800 languages and counting: lessons from survey research across a linguistically diverse continent. In Tracing Language Movement in Africa. [ EA Albaugh , KM de Luna , editors]. New York: Oxford University Press.

[19] Hentschel E , Yousafzai AK & Aboud FE (2021) The Nurturing Care Framework: Measuring Responsive Care and Early Learning Activities. Boston, MA: Harvard T.H. Chan School of Public Health.

[20] Kariger P , Frongillo EA , Engle P et al. (2012) Indicators of family care for development for use in multicountry surveys. J Health Popul Nutr 30, 472–486. 23304914 10.3329/jhpn.v30i4.13417 PMC3763619

[21] UNICEF (2021) Indicators for Assessing Infant and Young Child Feeding Practices: Definitions and Measurement Methods. New York: UNICEF.

[22] Abidin RR (2012) (PSI-4) Parenting Stress Index, 4th ed (PSI-4). Lutz, FL: Psychological Assessment Resources.

[23] UNICEF (2020) Questionnaire for children under five. Available at https://mics.unicef.org/tools?round=mics6 (accessed August 2023).

[24] Yalçin SS , Tezol Ö , Çaylan N et al. (2021) Evaluation of problematic screen exposure in pre-schoolers using a unique tool called “seven-in-seven screen exposure questionnaire”: cross-sectional study. BMC Pediatr 21, 472. 34696746 10.1186/s12887-021-02939-y PMC8546938

[25] Washington Group on Disability Statistics (2023) Available at https://www.washingtongroup-disability.com/ (accessed July 2023).

[26] Vazir S , Engle P , Balakrishna N et al. (2013) Cluster-randomized trial on complementary and responsive feeding education to caregivers found improved dietary intake, growth and development among rural Indian toddlers: responsive feeding, infant growth and development. Matern Child Nutr 9, 99–117. 22625182 10.1111/j.1740-8709.2012.00413.x PMC3434308

[27] Jeong J , Obradović J , Rasheed M et al. (2019) Maternal and paternal stimulation: mediators of parenting intervention effects on preschoolers’ development. J Appl Dev Psychol 60, 105–118.

[28] Forbes B , Fosuah C , Tidwell B et al. (2023) Effects of a nurturing care group behavior change program on child protection outcomes in Ghana: a controlled before and after trial. Child Abuse Negl 139, 106067. 36827866 10.1016/j.chiabu.2023.106067

[29] Gaidhane A , Telrandhe S , Holding P et al. (2022) Effectiveness of family-centered program for enhancing competencies of responsive parenting among caregivers for early childhood development in rural India. Acta Psychol (Amst) 229, 103669. 35878448 10.1016/j.actpsy.2022.103669

[30] Mehrin SF , Hamadani JD , Salveen NE et al. (2021) Adapting an evidence-based, early childhood parenting programme for integration into government primary health care services in rural Bangladesh. Front Public Health 8, 608173. 33537282 10.3389/fpubh.2020.608173 PMC7848202

[31] Walker SP , Baker-Henningham H , Chang SM et al. (2018) Implementation of parenting interventions through health services in Jamaica. Vulnerable Child Youth Stud 13, 127–141.

[32] Ahun MN , Aboud F , Wamboldt C et al. (2023) Implementation of UNICEF and WHO’s care for child development package: lessons from a global review and key informant interviews. Front Public Health 11, 1140843. 36875409 10.3389/fpubh.2023.1140843 PMC9978394

[33] Aftab W , Rabbani F , Sangrasi K et al. (2018) Improving community health worker performance through supportive supervision: a randomised controlled implementation trial in Pakistan. Acta Paediatr 107, 63–71. 10.1111/apa.14282 30570797

[34] Lamstein S , Perez-Escamilla R , Koniz-Booher P et al. (2017) The Community Infant and Young Child Feeding Counselling Package in Kaduna State, Nigeria: A Mixed Methods Evaluation. Final Report. Arlington, VA: Strengthening Partnerships, Results, and Innovations in Nutrition Globally (SPRING) Project.

[35] Galvin L , Verissimo CK , Ambikapathi R et al. (2023) Effects of engaging fathers and bundling nutrition and parenting interventions on household gender equality and women’s empowerment in rural Tanzania: results from EFFECTS, a five-arm cluster-randomized controlled trial. Soc Sci Med 324, 115869. 37023660 10.1016/j.socscimed.2023.115869

[36] Singla DR , Kumbakumba E & Aboud FE (2015) Effects of a parenting intervention to address maternal psychological wellbeing and child development and growth in rural Uganda: a community-based, cluster randomised trial. Lancet Glob Health 3, e458–e469. 26144389 10.1016/S2214-109X(15)00099-6

[37] Iwo R , Ruiz-Casares M & Nazif-Muñoz JI (2023) The increasing prevalence of children home alone in Ghana: the importance of considering regional inequalities. Child Indic Res 16, 2013–2032. 37711231 10.1007/s12187-023-10038-w PMC10497642

[38] Abdullah A , Frederico M , Cudjoe E et al. (2020) Towards culturally specific solutions: Evidence from Ghanaian kinship caregivers on child neglect intervention. Child Abuse Rev 29, 402–15.

[39] Cooke E , Hague S & McKay A (2016) The Ghana poverty and inequality report: Using the 6th Ghana Living Standards Survey. Available at http://africainequalities.org/wp-content/uploads/2016/07/Ghana_Poverty_and_Inequality_Analysis_FINAL_Match_20161.pdf (accessed December 2023).

[40] Coope CM & Theobald S (2006) Children at risk of neglect: challenges faced by child protection practitioners in Guatemala city. Child Abuse Negl 30, 523–36. 16704877 10.1016/j.chiabu.2005.11.007

[41] Ruiz-Casares M & Heymann J (2009) Children home alone unsupervised: modeling parental decisions and associated factors in Botswana, Mexico, and Vietnam. Child Abuse Negl 33, 312–323. 19477517 10.1016/j.chiabu.2008.09.010

[42] Al-Hassan RM & Diao X (2007) Regional Disparities in Ghana: Policy Options and Public Investment Implications IFPRI Discussion Paper No. 00693. Washington, DC: International Food Policy Research Institute (IFPRI).

[43] Baumgartner JN , Ali M , Gallis JA et al. (2021) Effect of a lay counselor-delivered integrated maternal mental health and early childhood development group-based intervention in Northern Ghana: a cluster-randomized controlled trial. Glob Ment Health 8, e18. 10.1017/gmh.2021.15 PMC8157813 34104458

[44] Huang KY , Bornheimer LA , Dankyi E et al. (2018) Parental wellbeing, parenting and child development in Ghanaian families with young children. Child Psychiatry Hum Dev 49, 833–41. 29589228 10.1007/s10578-018-0799-3 PMC6126985

[45] Jeong J , Pitchik HO & Yousafzai AK (2018) Stimulation interventions and parenting in low- and middle-income countries: A meta-analysis. Pediatr 141, e20173510. 10.1542/peds.2017-3510 29500293

[46] Madlala S & Kassier S (2018) Antenatal and postpartum depression: effects on infant and young child health and feeding practices. S Afr J Clin Nutr 31, 1–7.

[47] Rochat TJ , Redinger S , Rozentals-Thresher R et al. (2019) Caring for the Caregiver: Implementer’s Guide. New York, NY: UNICEF.

[48] Rogers A , Obst S , Teague SJ et al. (2020) Association between maternal perinatal depression and anxiety and child and adolescent development: a meta-analysis. JAMA Pediatr 174, 1082–1092. 32926075 10.1001/jamapediatrics.2020.2910 PMC7490743

[49] Wemakor A & Mensah KA (2016) Association between maternal depression and child stunting in Northern Ghana: a cross-sectional study. BMC Public Health 16, 869. 27557725 10.1186/s12889-016-3558-z PMC4997709

