# Supplementary material for: Promoting responsive care and early learning practices in Northern Ghana: results from a counselling intervention within nutrition and health services
Source: Public Health Nutr. 2024 Feb 8;27(1):e77. doi: 10.1017/S1368980024000156 (PMC10966855; doi:10.1017/S1368980024000156)
Supplement: Aidam et al. supplementary material [file S1368980024000156sup001.docx]

# **SUPPLEMENTARY MATERIALS**

# **Supplementary Material 1: Program Monitoring Data of Individuals Reached in the VSLA Group Meetings and Children Reached with Nutrition Interventions**

| **Monitoring Indicators** | **July-Sept 2022** | **Oct-Dec 2022** | **Jan-March 2023** | **April-June 2023** |
| --- | --- | --- | --- | --- |
| Number of individuals participating in the VSLA group meetings |  | 2,181 | 2,152 | 2,152 |
| Number of children under 2 (0–23 months) reached with community-level nutrition interventions | 823 | 1,279 | 1,602 | 1,250 |
| Number of children under 5 (0–59 months) reached with nutrition-specific interventions |  | 1,279 | 1,696 | 1,321 |

**Supplementary Material 2: Unpaired Baseline and Endline Differences for Children 12–23 Months Old at Baseline or Endline**

| **Indicator** | **Baseline (n=66)** | | **Endline (n=151)** | | **Change Baseline to Endline** | |
| --- | --- | --- | --- | --- | --- | --- |
|  | % or Mean | (SD) | % or Mean | (SD) | Percentage point change or unit change | *p* value |
| Caregiver-child interactions that are responsive to the child's cues | 23.3 | (18.5) | 60.3 | (18.3) | 37.1 | <0.001 |
| Caregiver-child interactions that are initiated by the caregiver | 72.6 | (19.7) | 38.5 | (18.5) | -34.1 | <0.001 |
| Caregiver-child interactions that are negative | 4.1 | (9.5) | 1.1 | (4.9) | -3.0 | 0.003 |
| Caregiver-child interactions that are verbal | 46.3 | (14.1) | 45.0 | (11.8) | -1.3 | 0.486 |
| Children with whom adult household members have engaged in four or more activities | 24.2 |  | 62.3 |  | 38.1 | <0.001 |
| Number of activities with adult household members | 3.1 | (1.1) | 3.9 | (1.4) | 0.8 | <0.001 |
| Children with whom fathers have engaged in four or more activities | 1.5 |  | 7.3 |  | 5.8 | 0.087 |
| Number of activities with fathers | 0.8 | (1.1) | 0.9 | (1.4) | 0.1 | 0.341 |
| Children with whom mothers have engaged in four or more activities | 6.1 |  | 39.7 |  | 33.6 | <0.001 |
| Number of activities with mothers | 2.2 | (1.1) | 3.1 | (1.5) | 0.9 | <0.001 |
| Children who have three or more children’s books | 3.0 |  | 4.6 |  | 1.6 | 0.585 |
| Children who play with homemade toys | 31.8 |  | 74.2 |  | 42.4 | <0.001 |
| Children who play with household objects/objects found outside | 68.2 |  | 85.4 |  | 17.2 | 0.003 |
| Children who play with toys from a shop/manufactured toys | 40.9 |  | 53.6 |  | 12.7 | 0.084 |
| Children who play with two or more types of playthings | 42.4 |  | 76.2 |  | 33.8 | <0.001 |
| Number of stimulating engagement activities by a caregiver with a child from 12–23 months with objects (e.g., playthings) and/or people (adults and peers) | 5.1 | (2.8) | 7.7 | (3.0) | 2.6 | <0.001 |
| Parental Distress Subscale (PD) Score | 33.9 | (9.2) | 29.9 | (10.0) | -4.0 | 0.006 |
| Parent-Child Dysfunctional Interaction (P-CDI) Sub-Scale Score | 32.5 | (5.0) | 30.6 | (5.8) | -1.9 | 0.027 |
| Difficult Child (DC) Sub-Scale Score | 33.1 | (5.9) | 29.8 | (7.5) | -3.3 | 0.002 |
| Total Stress Score | 99.5 | (15.5) | 90.4 | (18.8) | -9.1 | 0.001 |
| Caregivers reporting high parental stress | 31.8 |  | 11.3 |  | -20.5 | <0.001 |
| Left alone in the past week | 45.5 |  | 41.7 |  | -3.8 | 0.609 |
| Left under the supervision of another child younger than 10 years of age in the past week | 31.8 |  | 43.7 |  | 11.9 | 0.100 |
| Left with inadequate supervision in the past week | 48.5 |  | 63.6 |  | 15.1 | 0.038 |
| Children 12–23 months who are achieving minimum dietary diversity | 56.1 |  | 72.8 |  | 16.7 | 0.015 |
| Children 12–23 months who are achieving minimum meal frequency | 78.8 |  | 66.2 |  | -12.6 | 0.063 |
| Children 12–23 months who are achieving minimum acceptable diet | 50.0 |  | 52.3 |  | 2.3 | 0.753 |

**Supplementary Material 3: Bivariate Associations of Prioritized Factors with Outcomes of Interest**

| **Factors** | Children with whom adult household members have engaged in four or more activities | Caregivers reporting high parental stress | Children left with inadequate supervision in the past week | Children 6–23 months who are achieving minimum acceptable diet | Number of caregiver-child interactions that were responsive | Number of stimulating engagement activities by a caregiver | Number of caregiver-child interactions that are negative |
| --- | --- | --- | --- | --- | --- | --- | --- |
|  | RR‡  95% CI | RR  95% CI | RR  95% CI | RR  95% CI | RD‡  95% CI | RD  95% CI | RD  95% CI |
| **Caregiver Education** | | | | | | | |
| None Completed | Ref | | | | | | |
| Primary Completed | 0.91  (0.69-1.19) | 1.82  (0.86-3.85)† | 1.2  (0.96-1.50) | 0.68  (0.41-1.12) | -0.71  (-7.06-5.65) | 0.28  (-0.72-1.27) | 0.94  (-0.68-2.56) |
| Secondary or Higher Completed | 1.06  (0.85-1.33) | 0.48  (0.14-1.63)† | 0.97  (075-1.26) | 0.98  (0.70-1.37) | -0.54  (-6.64-5.56) | 0.74  (-0.21-1.70) | 1.29  (-0.27-2.84) |
| **Child’s Father Lives in the Home** | | | | | | | |
| Yes | 0.88  (0.67-1.17) | 1.38  (0.35-5.46) | 0.93  (0.67-1.27) | 0.87  (0.52-1.44) | -1.14  (-9.41-7.14) | 0.37  (-0.93-1.68) | 1.09  (-1.04-3.21) |
| No | Ref | | | | | | |
| **Child Sex** | | | | | | | |
| Male | Ref | | | | | | |
| Female | 1.27  (1.04-1.55)* | 0.57  (0.26-1.21)† | 0.92  (0.75-1.13) | 1.37  (1.02-1.86)† | 1.24  (-3.78-6.26) | 0.03  (-0.76-0.82) | -0.9  (-2.19-0.38)† |
| **Screen Exposure** | | | | | | | |
| Low | Ref | | | | | | |
| High | 0.90  (0.63-1.29) | 1  — †‡ | 1.13  (0.80-1.59) | 0.68  (0.34-1.37) | 5.13  (-3.64-13.90) | 0.21  (-1.14-1.56) | -0.58  (-3.36-2.20) |
| **Child’s Age** | 1.02  (1.01-1.04)* | 1.01  (0.94-1.09) | 1.01  (1.00-1.03)† | 1.02  (0.98-1.06) | 0.45  (0.02-0.89)* | 0.08  (0.01-0.14)* | -0.04  (-0.15-0.07) |
| **Mother’s Age** | 1.01  (0.99-1.02) | 1.03  (0.99-1.08)§ | 1.00  (0.99-1.02) | 0.99  (0.97-1.02) | -0.24  (-0.65-0.17) | 0.01  (-0.05-0.08) | -0.1  (-0.20-0.01)† |
| **Number of Household Members** | 0.99  (0.98-1.00)* | 0.94  (0.89-1.00)† | 1.01  (1.00-1.02) | 0.97  (0.95-1.00)* | 0.18  (-0.11-0.47) | -0.08  (-0.12-0.03)* | -0.02  (-0.09-0.56) |

* Statistically significant at p<0.05

† Statistically significant at p<0.20 for inclusion in multivariate regressions

‡ RR, Relative Risk; RD, Risk Difference

§ All caregivers who reported high parental stress reported their child having low screen exposure
